# Validating a Patient-Reported Outcome Measure to Improve Emergency Department Asthma Care: Protocol for an Observational Study

**DOI:** 10.2196/67195

**Published:** 2025-05-29

**Authors:** Michelle P Lin, Lauren Gordon, Lynne D Richardson

**Affiliations:** 1 Department of Emergency Medicine Stanford University Palo Alto, CA United States

**Keywords:** asthma, patient-reported outcomes, emergency care

## Abstract

**Background:**

Asthma affects 1 in every 12 persons in the United States, resulting in 1.9 million emergency department (ED) visits annually. However, the lack of patient-reported outcome measures (PROMs) validated for use in the ED limits the evaluation of interventions to improve ED asthma care.

**Objective:**

To address this knowledge gap, this study protocol will (1) develop and test the validity and reliability of the Patient Reported Outcomes for Acute Asthma Care and Treatment instrument (PROAACT), (2) test whether receiving more guideline-concordant ED care is associated with improved PROAACT responses, and (3) evaluate the association between PROAACT score and subsequent ED revisits and hospitalizations.

**Methods:**

This is a prospective cohort study of adult patients visiting the ED for acute asthma exacerbation across 3 EDs at an urban, tertiary care health system. Eligible patients are 18 years or older, have a prior diagnosis of asthma (self-reported or documented in the electronic health record), are English-speaking, and experiencing an ED visit for asthma exacerbation as determined by the treating clinician. Enrolled participants complete an initial PROM survey during their ED visit assessing their symptoms in the preceding 7 days, then complete a follow-up survey 7 days after ED discharge assessing changes in the symptoms in the subsequent 7 days. To test whether guideline-concordant care is associated with improved PROAACT scores, we will conduct a retrospective chart review of medications ordered during the ED visit, and then compare guideline adherence to changes in PROAACT scores. To test whether improved PROAACT scores are associated with fewer return ED visits and hospitalizations, we will extract all-cause ED visits and hospitalizations within 30 days from a regional health information exchange, and then compare usage to changes in PROAACT scores. We will use item response theory to develop scale responses based on summed item responses, which will allow us to test associations with clinical outcomes, including adherence to guideline-recommended care and return ED visits and hospitalizations.

**Results:**

Recruitment is ongoing and has experienced numerous challenges related to the COVID-19 pandemic. To date, we have enrolled over 250 participants and have completed over 200 follow-ups. Recruitment is expected to conclude in spring 2025.

**Conclusions:**

Our study is intended to validate the use of PROMs during ED visits for acute asthma exacerbation among adult patients. Completion of the proposed aims will result in one of the first PROMs intended for use among adult ED patients and support the feasibility of collecting PROMs in the ED setting.

**Trial Registration:**

Clinical Trials.gov NCT04349020; https://clinicaltrials.gov/study/NCT04349020

**International Registered Report Identifier (IRRID):**

DERR1-10.2196/67195

## Introduction

Asthma affects 1 in every 12 persons in the United States [1], resulting in 1.9 million emergency department (ED) [2] visits annually; however, the impact of ED care on patient-reported outcomes after acute exacerbations is unknown. Racial and ethnic minorities and individuals with low socioeconomic status are more likely to have asthma and experience worse health outcomes, including asthma-related ED visits and hospitalizations [3]. According to the Centers for Disease Control and Prevention (CDC), female, Black and Hispanic patients with asthma are more likely to seek care in the ED, be hospitalized, and die of asthma-related complications [4,5]. Thus, patients who seek asthma care in the ED represent a population in critical need of interventions to improve clinical and patient-reported outcomes.

Patient-reported outcome measures (PROMs) are surveys that have been developed to ensure valid and reliable measurement of these patient-reported outcomes [6]. The National Institutes of Health and National Quality Forum have endorsed the use of PROMs to assess health outcomes and quality of care [6-8]. However, the adoption of PROMs in clinical settings remains limited, in part due to the lack of PROMs validated for use in ED settings [9]. Existing PROMs assess disease control and outpatient disease management [10-15]; however, they have not yet been validated or used among patients seeking ED care for acute asthma exacerbations. Existing PROMs such as the Asthma Control Test (ACT) [12] and Asthma Quality of Life Questionnaire (AQLQ) [10] primarily assess disease control, or specific constructs such as quality of life, and were intended for—and thus developed exclusively in—clinical trials and outpatient disease management.

To assess this knowledge gap, we developed the Patient Reported Outcomes for Acute Asthma Care and Treatment (PROAACT) survey to evaluate PROMs among patients seeking care for acute asthma exacerbations in the ED. This study builds on our preliminary work of assessing the feasibility of administering a PROM in the ED and the ability to engage ED patients in PROM development [9].

**Figure 1:**
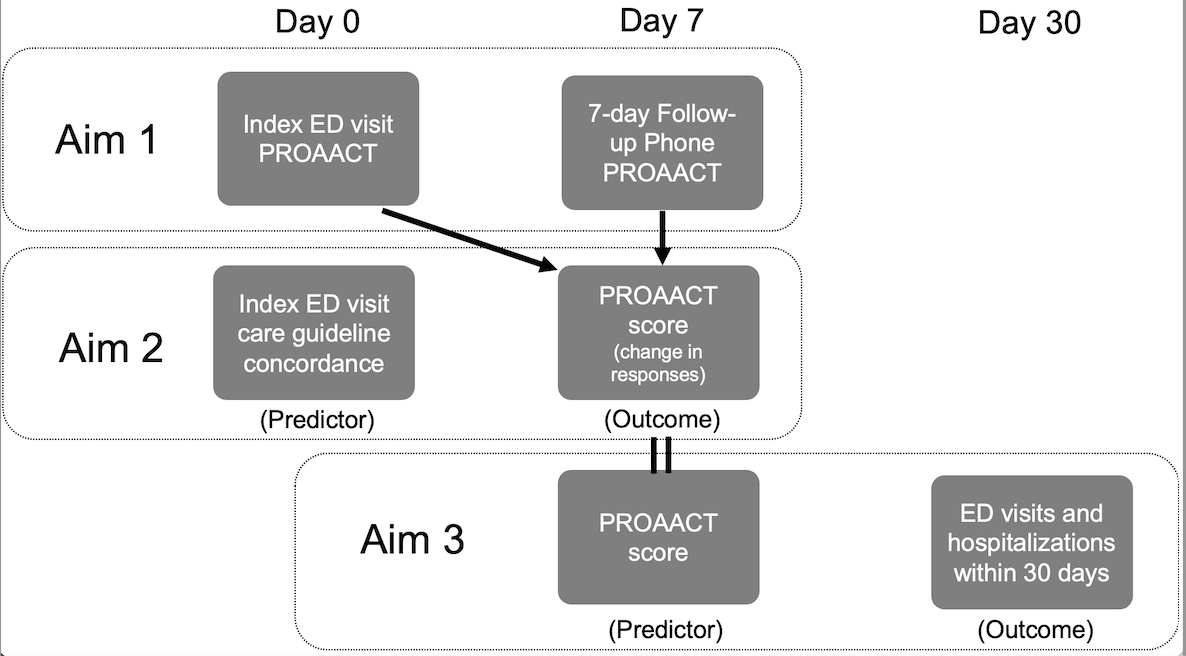
Data collection, outcomes, and predictors of interest. ED: emergency department; PROAACT: Patient Reported Outcomes for Acute Asthma Care and Treatment instrument.

Our study has three aims: (1) we will first assess the psychometric validity and reliability of the PROAACT survey to assess outcomes prioritized by adult ED patients with asthma; (2) then, we will test whether receiving more guideline-concordant ED care is associated with improved PROAACT responses 7 days after ED discharge; (3) and finally, we will evaluate the predictive validity by testing the association between PROAACT responses and 30-day ED revisits and hospitalizations (Figure 1).

## Methods

### Study Design

**Figure 2:**
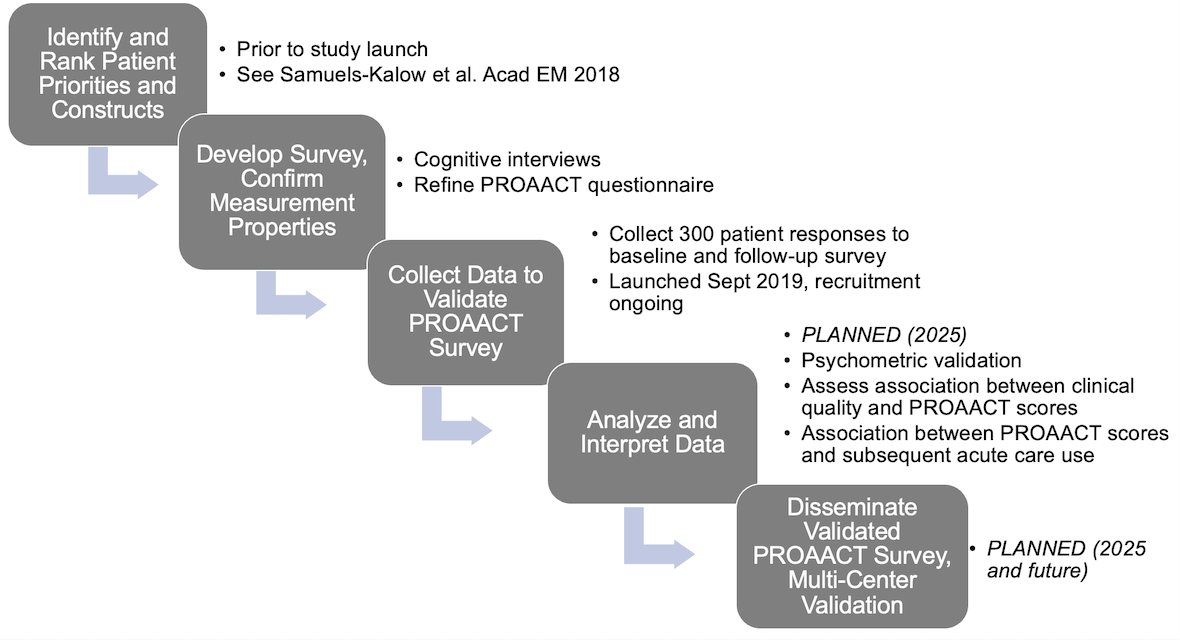
Study workflow and timeline [16]. PROAACT: Patient Reported Outcomes for Acute Asthma Care and Treatment instrument.

This is a prospective cohort study enrolling a convenience sample of adult patients experiencing an ED visit for acute asthma exacerbation. This study is funded by the National Heart, Lung, and Blood Institute, and the grant review summary statement is included in the Multimedia Appendix 1. We registered the trial at ClinicalTrials.gov (NCT 04349020). We developed questions to be used in the PROAACT questionnaire based on prior published work [16] identifying outcomes of key importance to ED patients experiencing asthma exacerbation (Figure 2). After reviewing PROMs assessed in asthma studies and related constructs, we updated the item set by mapping ranked constructs to previously validated Patient Reported Outcome Measurement Information System (PROMIS) [17] item banks and Consumer Assessment of Healthcare Providers and Systems (CAHPS) [18] Survey (Table 1). The complete baseline and follow-up PROAACT surveys are included in the Multimedia Appendix 2.

**Table 1:** Key patient-reported outcome measurement constructs, patient priority ranking, and validated question source.

Domain and ranking			Construct		PROMIS^a^ short form	
**Access to care**						Consumer Assessment of Health Care Provider and Systems (CAHPS)^b^
	1	Able to get medication supplies				
	6	Follow-up with primary care doctor				
**Dyspnea and symptoms**						
	2	Breathing better, less wheezing or coughing		Dyspnea severity		
**Self-efficacy**						
	3 (tie)	Taking medications right		Self-efficacy for managing medications		
	5	Knowing enough about asthma and your plan		Self-efficacy for managing symptoms		
**Ability to participate in activities**						
	3 (tie)	Return to normal activities/Sleeping well		Ability to participate in social roles and activities		
	7	Return to work or school		Satisfaction with participation in social roles		
**Anxiety and stress**						
	8	Not needing to go back to the ER		CAHPS		
		Feeling less worried or stressed		Dyspnea emotional response		

^a^PROMIS: Patient Reported Outcome Measurement Information System.

^b^Not PROMIS Short Forms.

### Study Population and Setting

Participants are eligible to be approached for the study if they are 18 years or older, English Speaking, with a respiratory complaint (eg, “shortness of breath” or “wheezing”), and a self-reported or documented past medical history of asthma, and experiencing an ED visit for asthma as per the treating ED clinician. Because this project is intended to focus specifically on outcomes after ED care, eligible patients include those intended to be discharged to home after the current ED visit for asthma. Patients who are admitted to the hospital after ED evaluation and treatment receive further treatment from inpatient providers and specialists; thus, PROMs in these conditions reflect the entire acute care episode, not solely care provided in the ED.

#### Exclusion Criteria

Participants who speak a language other than English are not included in the study due to the lack of validation of the study survey in other languages. Participants admitted to the hospital were also excluded from the study. Other exclusions are inability to consent (eg, due to dementia, psychosis, altered mental status, intoxication, hemodynamic instability), serious diagnosis identified in the ED (eg, clinically significant dysrhythmia, structural heart disease, gastrointestinal hemorrhage, myocardial infarction, pulmonary embolism, aortic dissection, ectopic pregnancy, subarachnoid hemorrhage, or stroke), pregnancy, acute COVID-19 infection (probable or confirmed), either symptomatic within 10 days of onset or asymptomatic within 5 days of a positive test and prior diagnosis of chronic obstructive pulmonary disease, inability to read in English (if a friend or family member can read follow-up survey questions, they may still be eligible; however, if they cannot complete the follow-up survey, they are ineligible for the study). Additional exclusions included lack of phone number or mailing address including zip code, email addresses, visually or hearing impaired, and persons in law enforcement custody due to inability to collect 7-day postdischarge surveys.

#### Setting

Participants are identified and enrolled at 3 urban academic EDs. Site 1 is a 1171-bed tertiary- and quaternary-care teaching facility with approximately 110,000 annual ED visits. Site 2 is an 856-bed teaching hospital with approximately 100,000 annual ED visits. Site 3 is a 495-bed hospital serving 130,000 annual ED visits. The racial and ethnic distribution of the patient population served by these EDs is 35% Hispanic, 29% African-American, 19% White, 1% Asian, and 16% other. With respect to insurance, 13% of adult patients had no insurance, 42% had Medicaid, 24% had private insurance, and 21% had Medicare.

#### Aim 1: Prospective Recruitment

Study data are collected and managed using REDCap (Research Electronic Data Capture; Vanderbilt University) [19,20] electronic data capture tools. REDCap is a secure, web-based software platform designed to support data capture for research studies, providing (1) an intuitive interface for validated data capture, (2) audit trails for tracking data manipulation and export procedures, (3) automated export procedures for seamless data downloads to common statistical packages, and (4) procedures for data integration and interoperability with external sources.

A trained research coordinator screens participants for eligibility using the electronic health record (EHR). Participants are recruited in the ED from 8 AM to 8 PM, Monday through Friday, during their ED visit for asthma and prior to discharge from the ED. After screening for eligibility criteria (age and presenting problem), the research coordinator approaches an ED clinician (physician, midlevel, or nurse) to confirm if the participant is proficient in English, being treated for asthma, and is appropriate to participate in a survey study.

After confirming eligibility with the clinician, the research staff approaches patients in person to explain the study purpose and what study enrollment entails, including the benefits and risks of participation. Written informed consent is then obtained by the research coordinator to participate in the survey. We use the electronic study database REDCap to capture electronic consent. The e-consent function in REDCap allows us to create a template of the institutional review board (IRB)–approved consent forms, which participants can review and sign electronically, as well as receive a completed copy of the signed copy via email. e-Consent [21] may improve recruitment and retention in clinical research studies by addressing cultural and literacy barriers by including optional explanatory material (eg, defining terms by hovering over them with the cursor). After documenting informed consent, a paper document outlining the purpose of the study, the requirements, risks, and benefits are given to all potentially interested participants to review. Patients are reminded that their clinical care is in no way affected by their acceptance or refusal to participate in the study.

#### Data Collection

Data are collected and stored in REDCap. After obtaining consent, participants are encouraged to complete the initial PROAACT survey questions by entering responses into the REDCap questionnaire via iPad. Research coordinators are available to assist those with limited literacy and are available to clarify survey items or read survey questions verbatim as needed by participants. Research coordinators then enter name, phone number, email, medical record number, and demographic information as per participant self-report (gender, race, ethnicity, educational attainment, and employment status). Research coordinators also document the extent to which they assisted participants with the completion of the survey.

### Follow-Up Procedures

#### Overview

A total of 7 days after enrollment and completion of the PROAACT survey at the index ED encounter, participants are asked to self-administer a post–ED discharge follow-up survey using an individualized REDCap survey linked to each participant. Research coordinators contact participants via email, phone, and text message using the contact information they provided at the time of enrollment. Emails and text messages are sent once daily for up to 3 consecutive days starting on day 6 to remind participants of their 7-day follow-up survey. Text messages are sent through Twilio (Twilio Inc) [22] for automated SMS survey distribution and response collection, a third-party web service that is integrated with REDCap, allowing users to send survey invitations and alerts to participants as SMS text messages. Research staff also attempted to call subjects a maximum of 3 times using on-site telephones in the ED research office. Upon establishing contact, subjects are reminded of the study and asked if they are able to complete the telephone survey.

Upon completion of the telephone survey, subjects receive a US $20 electronic or mailed gift card as an incentive. Subject names and mailing addresses are written directly onto envelopes to be mailed and are not stored. Participants may decline or withdraw from the study by written or verbal request; however, they do not receive the incentive if they elect to withdraw.

#### Aims 2 and 3: Assessing Concordance

To assess the association between PROAACT responses and adherence to guideline-recommended ED care, we will perform a structured extraction of EHR data at the conclusion of enrollment using the institutional data warehouse. We will collect patient-level factors, including age, sex, past medical history, medication history (to assess asthma severity), insurance status, smoking history, vital signs at triage, medications administered in the ED, and medications prescribed upon ED discharge.

We will obtain data on repeat ED visits and hospitalizations from a regional health information exchange. Prior studies have demonstrated that over 1 in 3 patients who have a repeat ED visit after discharge visit a different facility [23]. Among patients who frequently visit the ED (≥4 ED visits in 30 days), HIE (health information exchange) data from the Healthix Regional Health Information Organization (RHIO) data identified 16% more ED visits than single-site data [24]. Healthix data will be linked to PROAACT responses and patient-level variables collected from the EHR using the patient name and date of birth.

### Analysis

#### Overview

We plan to administer the baseline and follow-up PROAACT survey to 300 adult ED patients with asthma. Based on historical data on annual ED visits by adult patients for asthma across all sites, we estimate a total population size of approximately 4500 over 3.5 years. Thus, a sample of 300 subjects will allow us to approximate a 5% margin of error within a 95% CI. In order to obtain 300 completed follow-up surveys, we anticipate needing to enroll 350 patients in the initial survey phase. Although a baseline sample of 300 is too small for evaluating measurement equivalence, it is adequate for selected latent variable models used in factor analyses [25].

#### Aim 1 Analysis

Preliminary analyses with item response theory are conducted to examine information functions and derive item parameters for comparison with other sociodemographic groups from data collected in future studies. We evaluate candidate items for item reduction to generate the item set that provides maximum test information at the mean responses, which serve as the final, short-form survey for subsequent external validation, dissemination, and implementation. As part of the psychometric analysis, we develop scale responses based on summed item responses. This enables us to examine construct, convergent, criterion, and predictive validity by examining associations with clinical outcome variables, including adherence to guideline-recommended care and return ED visits and hospitalizations.

#### Aim 2 Analysis

Next, we will test the hypothesis that improved PROAACT responses are associated with guideline-adherent ED care. We will assess concordance with Level A GINA (Global Initiative for Asthma) recommendations [26]: (1) treatment with inhaled β-agonists in the ED, (2) treatment with systemic corticosteroids in the ED for patients who do not respond completely to initial short-acting β-agonist therapy, (3) treatment with inhaled corticosteroids in the ED for those not receiving systemic corticosteroids, and (4) not treating with intravenous β-agonists in the ED. Given prior research suggesting high adherence to some guidelines (eg, inhaled β-agonists), we will examine concordance as a composite binary predictor (adherence to all 4 guidelines vs not) and separately, evaluate each recommendation as an independent predictor. We exclude the level A recommendation to administer magnesium for severe asthma exacerbations because patients with severe asthma are likely to be admitted and likely unstable to participate in a survey.

We will assess the PROAACT responses (primary outcome) using a distribution-based approach such as the paired *t* statistic to evaluate the change in responses in relation to the probability that the change occurred at random. We will calculate baseline descriptive statistics for patient characteristics (sex, age, race, insurance, and burden of comorbid illness). We will analyze guideline concordance of ED care both for all patients and stratified by patient characteristics (sex, age category, race, insurance, and burden of comorbid illness), ED provider, and enrollment site in order to identify potential baseline disparities by patient characteristics, provider or institutional adherence to guidelines. We will analyze the relationship between guideline concordance of ED care and PROAACT responses in a multivariable regression applying generalized hierarchical mixed-effects models to account for repeated measures of the same patient, as well as within-provider and within-hospital correlation of observed outcomes. We anticipate a sample size of 300 with an α level of .05 and 80% power will detect a 12% difference in PROAACT responses between groups receiving guideline-concordant care versus not.

#### Aim 3 Analysis

To test our hypothesis that patients who report greater improvement in patient-reported outcomes after ED discharge will have fewer ED visits and hospitalizations during the ensuing 30 days. The primary outcome is the rate of acute care encounters (all-cause ED revisit or hospitalization) within 30 days after the index ED discharge. We anticipate an overall event rate of 30%-40% based on the observed statewide rate of 26% in our preliminary data and the higher prevalence of risk factors for increased asthma usage in our patient population (Black and Hispanic race, exposure to urban pollution including indoor pests and allergens, limited health care access, lower income). ED revisits resulting in admission will count as one event. Secondary outcomes include all-cause ED revisits and hospitalizations within 180 days. We use all-cause ED revisits or hospitalizations given our preliminary data showing a substantial majority of repeat ED revisits and admissions were asthma-related in New York State in 2013 and because the reliability of Healthix diagnosis codes varies by institution. While a sample size of 300 and 35% event rate may not be sufficient to quantify the magnitude of the relationship between PROAACT responses and subsequent health care usage, our test for association and directionality will support the predictive and longitudinal validity of PROAACT as a stand-alone measure of asthma quality.

We will calculate 30-day event rates (for ED revisits and hospitalizations) using each patient’s index ED visit as the unit of analysis. We will first perform univariate analyses using chi-square tests to compare groups with and without improved PROAACT responses. To test the adjusted association between PROAACT responses and acute care use, we will conduct multivariable regression, using rate-based outcome distribution such as negative binomial or Poisson with zero inflation in a hierarchical model to account for patient- and hospital-level confounders, such as age, sex, race, ethnicity, burden of chronic conditions, and site of index ED visit.

### Ethical Considerations

This study was approved by the Mount Sinai IRB (Mount Sinai IRB protocol number 19-00364 and Stanford IRB protocol number 66322). The IRB has determined that this research involves no greater than minimal risk. Minimal risk means that the probability and magnitude of harm or discomfort anticipated in the research are not greater in and of themselves than those ordinarily encountered in daily life or during the performance of routine physical or psychological examinations or tests (45CFR.46.102; 21CFR50.3k). The IRB approved this research under expedited review procedure categories 5 and 7. Study participants provided written informed consent. Participant data are secure and confidential in an encrypted and password-protected REDCap database. Data include identifiers to link PROAACT survey responses to usage data regarding subsequent ED revisits. Participants were paid US $20 via gift card upon completion of the follow-up survey.

## Results

Funding was received on August 15, 2019, and recruitment was launched in September 2019. As of December 2024, we have enrolled over 200 participants toward our goal of 300. Recruitment is expected to conclude in spring of 2025 and we anticipate peer-reviewed publication of our findings in early 2026.

## Discussion

### Overview

Although PROMs are necessary to evaluate the impact of ED Asthma care, no PROMs have been developed or validated in adult ED patients with asthma to date. To our knowledge, this study will be among the first PROMs validated specifically with ED patients for use in ED settings. The diverse population of patients presenting to the ED for asthma at our study sites has the potential to increase the generalizability of our findings.

We anticipate the successful completion of the PROAACT scale’s psychometric and predictive validity will enable its use to assess and improve the quality of ED care. The multidimensional nature of the PROAACT scale, which assesses not just physical symptoms such as dyspnea, but also perceptions of access to care, will enable targeted improvements to ED asthma care. Further, if we find patients who do not report improvement on the PROAACT scale have received care that is not concordant with GINA guidelines, we could develop interventions to improve provider adherence to guidelines, for example, clinical decision support and audit-and-feedback. Similarly, if patients without improvement in PROAACT responses are shown to have a higher risk of subsequent ED visits, care management programs may target these individuals for more closely coordinated follow-up care after ED discharge.

Despite their importance and patient-centeredness, PROMs are difficult to collect in the ED. The ED is a fast-paced clinical environment that imposes time and workflow constraints. Many patients are acutely ill and suffer from impaired cognition, which limits their ability to provide reliable responses. Health conditions are often multifactorial and undifferentiated in the ED, which is less problematic for patients with a history of asthma who present with clinical symptoms of acute asthma exacerbation, but may result in the exclusion of patients with new-onset asthma who present with dyspnea and other respiratory symptoms. Many EDs are safety-net institutions with limited technology, poor integration of PROMs with technology systems such as EHRs, and lack of resources to act on PROM data. Our study is intended as a validation study; however, overcoming these logistic barriers is important to enable routine PROM collection in acute care settings.

This study specifically focuses on ED patients who are discharged so that PROMs reflect the care that was provided in the ED setting. Over 80% of adult patients with asthma are discharged nationally [24], thus, these patients represent the majority of patients presenting to the ED with asthma. While the inclusion of admitted patients may allow for higher clinical severity, patients who are admitted to the hospital receive interventions such as specialist consultation, respiratory therapy treatments, and other types of care that do not reflect the quality of care provided in the ED.

We also considered alternative time periods for the follow-up survey in 7 days; however, we sought to adapt the PROAACT items from previously validated sources whenever possible. Thus, because 7 days [27] as previously been used as a time period for assessing post–ED discharge outcomes and the PROMIS scales assessing key domains prioritized by patients (dyspnea, ability to participate in daily activities, etc) had previously been validated for use on a 7-day time scale, we proceeded with a 7-day interval to assess changes in responses.

### Limitations

Our study has several limitations. First, this study will be conducted in 3 urban, academic EDs in the Northeast, and the study results may not be generalizable to other EDs. However, we are enrolling a racially, ethnically, and socioeconomically diverse population that will provide useful insights for many clinical ED settings. Second, the PROAACT survey is not yet translated or validated in non–English-speaking populations; due to this, we have excluded non-English speakers. We anticipate including non-English speakers in a future study, which will require additional qualitative interviews and validation. Finally, recruitment was launched in late 2019 and impacted by the COVID-19 pandemic shortly thereafter, which has resulted in the following key modifications:

First, the recruitment period, initially intended to be 3.5 years in duration, has been extended to 5 years due to the COVID emergency pandemic occurring in March 2020. Recruitment for the study was paused from April through June 2020 coinciding with the initial surge of the COVID-19 pandemic in New York City. Active recruitment resumed in July 2020 and has been actively screening and enrolling patients since then; however, total ED visit volumes continued to be lower than pre–COVID-19 levels, consistent with national trends showing reductions of up to 42% compared to 2019 [28]. While ED visit volumes have since increased in each subsequent year, they have not yet returned [29,30] to pre–COVID-19 levels. Thus, recruitment is still ongoing. We also experienced substantial turnover in staff, which delayed our ability to recruit eligible patients. These workforce changes are consistent with national trends showing double the historical rate of job resignations in 2021 [29,31]. To fill this gap, we used medical students and undergraduate students to supplement recruitment needs.

### Conclusion

Our study is among the first PROMs developed specifically with ED patients for use in ED settings and has the potential to be used in future interventions to improve ED asthma care. We anticipate study results will inform a future study in which there will be a multicenter validation and implementation study of PROAACT. The validation of a PROM for ED asthma will enable the evaluation of interventions to improve ED asthma care and accelerate improvements in asthma care quality.

## Appendix

Multimedia Appendix 1 Peer review report from MPOR (MA) - NHLBI Mentored Patient-Oriented Research Review Committee (National Institutes of Health, USA).

Multimedia Appendix 2 PROAACT (Patient Reported Outcomes for Acute Asthma Care and Treatment instrument) survey.

## Abbreviations

ACT: Asthma Control Test
AQLQ: Asthma Quality of Life Questionnaire
CAHPS: Consumer Assessment of Healthcare Providers and Systems
CDC: Centers for Disease Control and Prevention
ED: emergency department
EHR: electronic health record
GINA: Global Initiative for Asthma
HIE: health information exchange
IRB: Institutional Review Board
PROAACT: Patient Reported Outcomes for Acute Asthma Care and Treatment instrument
PROMIS: Patient Reported Outcome Measurement Information System
PROM: patient-reported outcome measure
REDCap: Research Electronic Data Capture
RHIO: Regional Health Information Organization
