# Supplementary material for: Validating a Patient-Reported Outcome Measure to Improve Emergency Department Asthma Care: Protocol for an Observational Study
Source: JMIR Res Protoc. 2025 May 29;14:e67195. doi: 10.2196/67195 (PMC12163351; doi:10.2196/67195)
Supplement: Multimedia Appendix 1 [file resprot_v14i1e67195_app1.pdf]

**1 K23 HL143042-01 LIN, MICHELLE**

**RESUME AND SUMMARY OF DISCUSSION:** This is a new K23 application from Dr. Michelle Lin in which she has proposed a study focused on patient-reported outcomes of patients with asthma seeking emergency medical care. Dr. Lin has a modest, but growing publication record which includes four first author publications. The Career Development Plan is tailored to her immediate training and research goals and is complementary to her previous training and skills. There was discussion that meetings with the mentors/advisors are too infrequent. The Mentors are outstanding; Dr. Richardson, her primary mentor, has trained four K awardees to independence. The Research Plan proposes to develop a patient-reported outcomes measure for patients with asthma visiting the ED, evaluate the association of patient-reported and clinical outcomes, and compare risk prediction models that include patient-reported outcomes and social determinants to predict ED visits. The research proposed has clinical relevance. There were some concerns that Aim 3 is underpowered. Overall, this is a strong application from a promising young investigator with ideal mentors to lead her to research independence. Some weaknesses in the Career Development Plan, plans for meeting with mentors, and the research plan lowered overall enthusiasm.

**DESCRIPTION (provided by applicant):** This K23 award will enable the candidate, Michelle Lin, MD, MPH, MS, to become an independent physician scientist focused on developing, measuring and improving patient- reported outcomes for emergency department (ED) care of asthma and other acute cardiopulmonary conditions. Asthma affects 1 in every 12 persons in the U.S., resulting in 1.9 million ED visits annually; however, the impact of ED care on patient-reported outcomes after encounters for acute exacerbations is unknown. Dr. Lin's proposed study aims to develop a patient-reported outcomes measure (PROM) for adult asthma patients seeking care in the ED, evaluate the association between patient-reported and clinical outcomes, and acquire skills to improve the risk adjustment of asthma-related health outcomes. To achieve these goals, this proposal includes an integrated curriculum consisting of intensive mentorship and didactic coursework in patient engagement and patient-centered research, psychometric methods, advanced statistical modeling, and geospatial techniques. Her development and training activities also include building a research network, engaging with asthma patients in local communities, and participating in national scientific meetings. Dr. Lin has assembled a mentorship team of national leaders with expertise in patient-oriented asthma and emergency medicine research, patient-centered outcomes research, healthcare quality measurement, geospatial methods, and risk adjustment. In her pilot work, Dr. Lin has identified candidate items and scales for the novel Patient-Reported Outcomes for Acute Asthma Care and Treatment (PROAACT) instrument. During this K23, Dr. Lin will 1) evaluate the validity and reliability of the PROAACT instrument and 2) determine whether changes in PROAACT responses are associated with clinical outcomes (receiving more guideline-concordant ED care and lower rates of subsequent acute care utilization) while 3) acquiring skills to improve outcome measurement by incorporating PROM and geocoded social risk factors into risk adjustment models. Completion of this proposed project will result in a novel PROM for adult ED asthma patients and advance the understanding of how ED care impacts patient-reported outcomes, which will inform next steps for developing and implementing PROMs in acute care settings. Completing the proposed project and didactic training plan will also facilitate Dr. Lin's transition to an independent clinical investigator focused on improving patient-reported outcomes for asthma and other cardiopulmonary conditions treated in ED settings.

**PUBLIC HEALTH RELEVANCE**

Project Narrative: Asthma affects 1 in every 12 persons in the U.S., resulting in 1.9 million ED visits annually; however, the impact of ED care on patient-reported outcomes after acute exacerbations is unknown. The proposed research will train a physician-scientist to develop a novel instrument to assess patient-reported outcomes after adult ED asthma visits, evaluate the association between patient-reported and clinical outcomes, and improve outcome measurement by acquiring advanced skills in risk adjustment methodology.

**CRITIQUE 1:**

Candidate: 2
Career Development Plan/Career Goals /Plan to Provide Mentoring: 4
Research Plan: 4
Mentor(s), Co-Mentor(s), Consultant(s), Collaborator(s): 4
Environment Commitment to the Candidate: 1

**Overall Impact:**
This is a first submission from a well-trained candidate who is seeking an independent research career studying patient-centered outcomes in asthma patients treated in emergency care. The candidate's background and publications record thus far are strong, demonstrating a commitment to research and a clear focus. There are moderate concerns regarding the career development plan and mentorship, given very little didactic training is proposed as well as infrequent meetings with some mentors or advisors (including some off-site). However, the primary mentor and co-mentor Dr. Schuur are very well qualified and have excellent track records in securing NIH funding and serving as mentor. The research plan will be a good training vehicle, however there were minor concerns that the environmental data (while interesting) may be an unnecessary distraction from the main objectives of the candidate. More details on plans for an R01 would be helpful. The environment is excellent and there is strong institutional commitment to the candidate.

**1. Candidate:**
**Strengths**
- MD/MHP from Northwestern, MS in Clinical Epi from Harvard.
- Health Policy fellowship at Brigham and Women's Hospital.
- Currently Assistant Prof at Icahn School of Medicine, Mt Sinai.
- Growing publications record, mostly during 2016-2017. Six publications total, 4 as first author.
- Research focus clearly defined and related to publications thus far.
- Has earned small grants from Emergency Medical Foundation and other organizations.

**Weaknesses**
- Fairly new to research, but has shown dedication by completing MS in clinical epidemiology.

**2. Career Development Plan/Career Goals & Objectives/Plan to Provide Mentoring:**
**Strengths**
- Seeks independent career in patient-reported outcomes following ED care for asthma and other cardiopulmonary conditions (including associations with clinical outcomes).
- Outlines new training requirements in patient-reported outcomes, including instrument development and validations, and risk adjustment/outcomes.
- Has experience working with primary mentor Dr. Richardson, as well as co-mentor Dr. Schuur from Harvard.
- Meetings with primary mentors are acceptable frequency, but some of the other content experts (e.g. Dr. Teresi, who is listed as advisor, has key expertise in instrument development) is remote and only monthly meetings are proposed.
- Large team of mentors and advisors in general; meetings with most of the advisors are infrequent (e.g. quarterly) and most are remote.

**Weaknesses**
- The only formal didactic training proposed is in geospatial (Urban Spatial Statistics) which seems less well integrated into the proposal.

**3. Research Plan:**
**Strengths**

- Primary Aim is to develop and validate the patient-reported outcome measures instrument (PROAACT) for patients with asthma who visit the ED.
- Aim 2 will examine how PROAACT relates to clinical outcomes, such as acute care visits over 30 days post-discharge.
- Aim 3 combines social factors (including age, sex, income, etc, as well as environmental factors such as air pollution levels) and PROs in a risk adjustment model for clinical outcomes.
- The candidate has already begun preliminary work on the key items to be included on the PRO instrument.
- Good access to patients.
- The research plan will provide experience with assessment of PRO in patients with asthma, as well as extraction of information from EMR and analysis in risk prediction models.

**Weaknesses**

- Total sample size of 200 will be underpowered for some of the aims; however, should provide useful preliminary data for future work.
- Subsequent R01 or PCORI study for “multi-center validation trial” of the PROAACT instrument needs further detail. For example, it is unclear if this will be a larger-scale extension of what is proposed in the K23, and what the proposed outcomes would be. It is difficult to evaluate whether this will serve as a good transition to independence.
- The air pollution data integration (through geocoding) seems beyond the scope of “social factors” for risk prediction.

**4. Mentor(s), Co-Mentor(s), Consultant(s), Collaborator(s):**

**Strengths**

- Lynne Richardson, the primary mentor, is well funded and has appropriate expertise in emergency medicine. She has considerable mentoring experience through her involvement in K12 in Emergency Medicine and T32. She has worked with the candidate.
- Dr. Schuur worked with the candidate at Harvard and will continue to serve as mentor with expertise in emergency medicine and patient-reported outcomes.

**Weaknesses**

- Although the primary mentor is local, there is a large team of mentors and advisors including many who are off-site. Meetings with some are infrequent, including Dr. Teresi who has key expertise in instrument development (monthly meetings).
- Weekly meetings with primary mentor would provide more cohesiveness during training, especially since there is little formal coursework to be done.

**5. Environment and Institutional Commitment to the Candidate:**

**Strengths**

- Resources at the Icahn School of Medicine are excellent, including informatics and research, a large emergency department, and training in clinical research.
- Strong letter from the institution, assuring 75% protected time for the candidate.

**Weaknesses**

- None

**CRITIQUE 2:**

Candidate: 3
Career Development Plan/Career Goals /Plan to Provide Mentoring: 1
Research Plan: 4
Mentor(s), Co-Mentor(s), Consultant(s), Collaborator(s): 1
Environment Commitment to the Candidate: 1

**Overall Impact:**
High level of enthusiasm for this initial K23 application for career development and mentored research in patient-oriented asthma ED outcomes. Well-thought out application that is likely to lead to independence. Major strengths include the mentors, environment, and career development plan. The candidate is also outstanding, although productivity (as measured by publications) is modest for someone 5 years out of training. The research plan is also largely well developed and seems to be an excellent training tool that will also yield important data for future research. There are some minor weaknesses to the research plan that diminish enthusiasm. Overall, highly likely that the candidate will transition to independence.

**1. Candidate:**
**Strengths**
- Asst Prof of Emergency Medicine at Mount Sinai; finished fellowship 5 years ago.
- MPH in Public Health during med school + MS in Clinical Epidemiology at Harvard SPH during a Health Policy Research fellowship at Brigham.
- Career goal: “seeks to improve patient-centered outcomes for the emergency care of asthma and other acute cardiopulmonary conditions.” Current goal: “My goal is to acquire skills in patient-oriented research in order to become an independent clinical investigator with expertise in measuring and improving patient-reported outcomes and disparities in care for asthma and other acute cardiopulmonary conditions commonly treated in the ED”.
- Multiple prior awards and grants as PI: ABMS Visiting Scholar, B-CSRIP, Emergency Medicine Foundation Health Policy Research Scholar Award.
- 3 first author research publications + 1 other publication.
- CMS consultant; numerous awards and honors.
- Letters describe a spectacular candidate with tremendous potential for success.

**Weaknesse**s
- Publication record is a bit modest for 5 years out from fellowship.

**2. Career Development Plan/Career Goals & Objectives/Plan to Provide Mentoring:**
**Strengths**
- Worked with mentors to develop a plan that fills knowledge/skills gaps and builds upon prior training and experience: Patient-oriented ED & asthma research, instrument development and validation, risk adjustment.
- Each module is tied to a mentor and to the research plan and to the candidate’s goals.
- Detailed activities described (mentor meetings/calls, seminars/courses).
- Deliverables clearly delineated.

**Weaknesses**
- None

**3. Research Plan:**
**Strengths**
- Aims: develop a novel PRO asthma outcome measure, examine PRO outcomes and clinical outcomes, combine PRO with social determinants of health to predict ER use.
- Prelim data identifying constructs for the PROM; hospital-level variation in adult ER asthma visits.
- Mixed methods approach appears to be largely rigorous.

**Weaknesses**
- Would have liked to have seen more detail about how multivariable modeling will be performed in Aims 2a and 2b (e.g., potential confounders; how variables will be selected for the models).
- While risk-adjustment is important and social determinants of health are underrepresented in risk-adjustment, the logic of Aim 3 is not clear. Why are you combining PROM, social

determinants, and other clinical/demographic variables? How does this tie into the rest of the research plan? Why hypothesize that PROM adds value? Isn't it more important that you predict well rather than have one measure improve prediction? Would also like to see additional details about the Aim 3 model construction. There are numerous ways to build these models. Analysis plan seems generic.

**4. Mentor(s), Co-Mentor(s), Consultant(s), Collaborator(s):**
**Strengths**
- Mentor: Lynne Richardson, Professor of Emergency Medicine and Population Health Science & Policy; Vice Chair, Department of Emergency Medicine, R01 & U01 clinical investigator. Previously mentored 4 K awardees to R-level funding. One current K mentee. Co-published 1 paper with the candidate (albeit not first/last). Highly qualified mentor.
- Dr. Schuur (risk adjustment; co-published with candidate as senior author twice; is at Brigham); Dr. Wisnivesky (asthma, research methods), Teresi (psychometrics), Carr (spatial methods).

**Weaknesses**
- None

**5. Environment and Institutional Commitment to the Candidate:**
**Strengths**
- Outstanding
- 75% protected time

**Weaknesses**
- None

**CRITIQUE 3:**

Candidate: 1
Career Development Plan/Career Goals /Plan to Provide Mentoring: 4
Research Plan: 4
Mentor(s), Co-Mentor(s), Consultant(s), Collaborator(s): 3
Environment Commitment to the Candidate: 3

**Overall Impact:**
Dr. Michelle Lin, an emergency medicine physician at the Icahn School of Medicine at Mount Sinai, is the PI for this K23 career development application, which is concerned with developing patient reported outcomes for emergency department care, particularly for adults with asthma (Patient-Reported Outcomes for Acute Asthma Care and Treatment (PROAACT)). The PI will: (1) develop patient-reported outcome measures specific to ED asthma care with a cohort of 200 patients having acute asthma exacerbation; (2) link electronic health data to patient-reported outcome measures to assess for associations to outcomes such as acute care utilization within 30 days; and (3) develop and compare prediction models for acute care utilization. In this last aim, the PI will incorporate geocoded variables such as census tract associated income and air quality. The candidate is strong with an excellent career trajectory and the mentorship team is also excellent and have already established a working relationship with the PI and with each other. There is some reduced enthusiasm with the research plan in regard to how the PI may account for hospitalized patients as well as some lack of clarity on how this proposal and career development plan will optimally position the PI to transition to an independent researcher.

**1. Candidate:**
**Strengths**

- The candidate has made progress in her academic career training, including MPH at Northwestern University, MS in epidemiology at Harvard School of Public Health, and a Health Policy Research Fellowship at Brigham and Women's. She is now Assistant Professor at Icahn.
- The candidate has demonstrated productivity thus far in her training in research, with 3 first author peer-reviewed publications in areas related to this proposal. She has also competed successfully for small grants as PI from the Emergency Medicine Foundation.

**Weaknesses**
- None noted.

**2. Career Development Plan/Career Goals & Objectives/Plan to Provide Mentoring:**
**Strengths**
- Coursework at the Columbia Mailman School of Public Health and NYU are proposed to enhance career development. The candidate already has a MPH degree and the proposed coursework and mentoring are designed to increase the PI's skills in risk adjustment techniques and other patient-oriented clinical research methods.
- The research progression via the aims in this project will lead to acquisition of methodologic skills for the PI which will be applicable to other areas of clinical inquiry, and thus foster career development.

**Weaknesses**
- Although the general plan for career development will certainly increase the PI's skills and experience, and also mention of a multicenter R01 further PROAACT validation, there is a lack of other specific details on the next steps that may follow after the completion of the K23. While there are statements on seeking further funding and general areas of inquiry, it is not clear how the current proposal may lead to either other related areas of inquiry in emergency medicine, or further probing of asthma specifically in the ED.
- In regard to the plan for a multicenter R01 as the next step / as well as some of the other future directions outlined, it is not clear that the career development plan as outlined will optimally prepare the PI for these directions.

**3. Research Plan:**
**Strengths**
- There is a strong case that patient-reported outcome measures for asthma in the ED would reflect the quality of care provided in the ED (and different than other conditions that more commonly lead to hospitalization).

**Weaknesses**
- Although there is a case made for current patient-reported measures being inadequate in the ED setting, it is unclear whether this is a specific gap in the ED only, or just a limitation of patient-reported measures in general for this condition.
- It seems that there may be a challenge in having enough patients for discriminating the instrument using the 30-day period for re-visit to the ED / there are not adequate justifications in regard to sample size. In regard to ED visit, it is unclear why the investigators have chosen as the primary outcome to be all-cause ED visit and/or hospitalization and not asthma-specific.
- Although there is rationale for excluding hospitalized patients as measures may reflect inpatient and ED care, it would seem that these would be the sickest patients and perhaps an important group to consider in evaluating care and outcomes. In this regard, a plan to account for this group, or consider how exclusion may impact the study design and interpretation, may strengthen the proposal.

**4. Mentor(s), Co-Mentor(s), Consultant(s), Collaborator(s):**
**Strengths**

- The primary mentor, Dr. Richardson, has a track record of research and mentoring productivity, with extramural funding support. She has been the Program Director of a K12 program and mentored several successful K awardees.
- Co-mentor Dr. Schuur at Brigham and Women's continues to be a mentor and has expertise in patient reported outcomes. The primary and co-mentor have a strong working relationship despite not being at same institution.
- There is a good complement of expertise with Dr. Wisnivesky serving as content expert in the area of adult asthma and self-management, Dr. Teresi serving as a consultant in the area of measurement, Dr. Carr and Dr. Egorova in the areas of modeling and risk adjustment methods, and Dr. Hess in the area of patient engagement and shared-decision making.

**Weaknesses**
- Although there is a strong overall mentorship team, there may be some gaps, specifically for Aim 3 in regard to the impact of the social and built environment on asthma related outcomes, as well as on patient-reported outcomes in general.

**5. Environment and Institutional Commitment to the Candidate:**
**Strengths**
- The Department of Emergency Medicine has a specific Research Division with its own chair (Lynne Richardson, who is also the primary mentor for this proposal). It is noted that due to it being a relatively young academic specialty, that the department has developed connections for collaboration with other departments. This Division has now received a variety of extramural funding, including from NIH. The Department hosts a K12 program specific to emergency medicine.
- The ED at Mount Sinai and affiliated hospital have an ample patient population to conduct this research proposal.

**Weaknesses**
- Not clear if there is a strong peer group in similar career progression as the PI and how that may facilitate the career progression of the PI.
- Although the group has worked on disparities and social risk and outcomes, there may be a gap in providing optimal mentorship on the complex relationships amongst the social and built environment, access, and health outcomes.

**THE FOLLOWING SECTIONS WERE PREPARED BY THE SCIENTIFIC REVIEW OFFICER TO SUMMARIZE THE OUTCOME OF DISCUSSIONS OF THE REVIEW COMMITTEE, OR REVIEWERS' WRITTEN CRITIQUES, ON THE FOLLOWING ISSUES:**

**PROTECTION OF HUMAN SUBJECTS (RESUME): ACCEPTABLE**

**INCLUSION OF WOMEN PLAN (RESUME): ACCEPTABLE**

**INCLUSION OF MINORITIES PLAN (RESUME): ACCEPTABLE**

**INCLUSION OF CHILDREN PLAN (RESUME): ACCEPTABLE; no children involved, scientifically justified.**

**TRAINING IN THE RESPONSIBLE CONDUCT OF RESEARCH: ACCEPTABLE**

**RESOURCE SHARING PLANS: NOT APPLICABLE (NO RELEVANT RESOURCES)**

**AUTHENTICATION OF KEY BIOLOGICAL AND/OR CHEMICAL RESOURCES: NOT APPLICABLE (NO RELEVANT RESOURCES)**

**COMMITTEE BUDGET RECOMMENDATIONS: RECOMMENDED AS REQUESTED**

---

Footnotes for 1 K23 HL143042-01; PI Name: Lin, Michelle

NIH has modified its policy regarding the receipt of resubmissions (amended applications). See Guide Notice NOT-OD-14-074 at http://grants.nih.gov/grants/guide/notice-files/NOT-OD-14-074.html. The impact/priority score is calculated after discussion of an application by averaging the overall scores (1-9) given by all voting reviewers on the committee and multiplying by 10. The criterion scores are submitted prior to the meeting by the individual reviewers assigned to an application, and are not discussed specifically at the review meeting or calculated into the overall impact score. Some applications also receive a percentile ranking. For details on the review process, see http://grants.nih.gov/grants/peer_review_process.htm#scoring.
