# Supplementary material for: Validating a Patient-Reported Outcome Measure to Improve Emergency Department Asthma Care: Protocol for an Observational Study
Source: JMIR Res Protoc. 2025 May 29;14:e67195. doi: 10.2196/67195 (PMC12163351; doi:10.2196/67195)
Supplement: Multimedia Appendix 2 [file resprot_v14i1e67195_app2.pdf]

PROACT Survey

Please complete the survey below. The instructions for each section appear in BLUE.

Thank you!

Please indicate how you feel RIGHT NOW compared how you felt before your ED visit

	Not at all	A little bit	Somewhat	Quite a bit	Very much
My symptoms have improved, for example better breathing, less wheezing, or less coughing	○	○	○	○	○
I am able to return to my normal activities, including work or school	○	○	○	○	○
I will be able to sleep better	○	○	○	○	○
I am confident I am able to follow-up with primary care doctor	○	○	○	○	○
I am being prescribed the correct medications and supplies	○	○	○	○	○
I am taking my medications right	○	○	○	○	○
I am able to afford medications and supplies	○	○	○	○	○
I know enough about asthma	○	○	○	○	○
I know what to do if my asthma symptoms get worse	○	○	○	○	○
I feel less worried about my asthma	○	○	○	○	○
I am confident I will not need to go back to the ER	○	○	○	○	○

Please consider the NEXT 7 days and indicate:

	Not at all	A little bit	Somewhat	Quite a bit	Very much
In the next 7 days, I anticipate difficulty scheduling a follow up appointment with my primary care or asthma provider (for any reason, not necessarily related to asthma)	○	○	○	○	○
In the next 7 days, I anticipate difficulty attending a follow up appointment with my primary care or asthma provider (for any reason, not necessarily related to asthma)	○	○	○	○	○

In the next 7 days, I anticipate difficulty obtaining my prescribed medications	◯	◯	◯	◯	◯
In the next 7 days, I anticipate difficulty affording for my prescribed medications	◯	◯	◯	◯	◯

Please consider the LAST 7 days and indicate:

	Not at all	A little bit	Somewhat	Quite a bit	Very much
In the last 7 days, how often was it easy to get the care, tests, or treatment you needed? (for any reason, not necessarily related to asthma)	◯	◯	◯	◯	◯
In the last 7 days, how often were you unable to see a primary care doctor for your asthma because of the cost?	◯	◯	◯	◯	◯
In the last 7 days, how often were you referred to a specialist for asthma care but could not go because of the cost?	◯	◯	◯	◯	◯
In the last 7 days, how often was it easy to get the medication you needed?	◯	◯	◯	◯	◯
In the last 7 days, was there a time when you needed to buy medication for your asthma but could not because of the cost?	◯	◯	◯	◯	◯

Please indicate your current level of confidence

	I am not at all confident	I am a little confident	I am somewhat confident	I am quite confident	I am very confident
I can follow directions when my doctor changes my medications	◯	◯	◯	◯	◯
I can take my medication when there is a change in my usual day (unexpected things happen)	◯	◯	◯	◯	◯
I can manage my medications without help	◯	◯	◯	◯	◯
I can list my medications, including the doses and schedule	◯	◯	◯	◯	◯

Please indicate your current level of confidence

	I am not at all confident	I am a little confident	I am somewhat confident	I am quite confident	I am very confident
I can manage my symptoms during my daily activities	○	○	○	○	○
I can keep my symptoms from interfering with relationships with friends and familty	○	○	○	○	○
I can manage my symptoms in a public place	○	○	○	○	○
I can work with my doctor to manage my symptoms	○	○	○	○	○

50% Percent Complete
.

Over the past 7 days, how short of breath did you get with each of these activities?

	No shortness of breath	Mildly short of breath	Moderately short of breath	Severely short of breath	I did not do this in the past 7 days
Dressing yourself without help	○	○	○	○	○
Walking 50 steps/ paces on flat ground at normal speed without stopping (approx 1/2 street block)	○	○	○	○	○
Walking up 20 stairs (2 flights) without stopping	○	○	○	○	○
Preparing meals	○	○	○	○	○
Washing dishes	○	○	○	○	○
Sweeping or mopping	○	○	○	○	○
Making a bed	○	○	○	○	○
Lifting something weighting 10-20 lbs (about 4.5-9kg, like a large bag of groceries)	○	○	○	○	○
Carrying something weighing 10-20 lbs (about 4.5-9kg, like a large bag of groceries) from one room to another	○	○	○	○	○
Walking (faster than your usual speed) for mile (or 10 street blocks) without stopping	○	○	○	○	○

In the PAST 7 days

	Not at all	A little bit	Somewhat	Quite a bit	Very much
I worry about becoming short of breath	○	○	○	○	○
I become afraid when I have trouble catching my breath	○	○	○	○	○
Exercise seems unsafe for me	○	○	○	○	○
I am embarrassed by using my inhaled medication in public	○	○	○	○	○
I am embarrassed by using my oxygen in public (skip if you don't use oxygen)	○	○	○	○	○
I am embarrassed by my shortness of breath	○	○	○	○	○
I get upset when I can't do something because of my shortness of breath	○	○	○	○	○

75% Percent Complete
.

In the PAST 7 days

	Never	Rarely	Sometimes	Usually	Always
I have trouble doing all of my regular leisure activities with others (ex. social events)	○	○	○	○	○
I have trouble doing all of the family activities that I want to do (ex. Playing with children)	○	○	○	○	○
I have trouble doing all of my usual work (include work at home)	○	○	○	○	○
I have trouble doing all of the activities with friends that I want to do	○	○	○	○	○

In the PAST 7 days

	Not at all	A little bit	Somewhat	Quite a bit	Very much
I am satisfied with how much work I can do (include work at home)	○	○	○	○	○
I am satisfied with my ability to work (include work at home)	○	○	○	○	○

I am satisfied with my ability to do regular personal and household responsibilities	◯	◯	◯	◯	◯
I am satisfied with my ability to perform my daily routines	◯	◯	◯	◯	◯

95% Percent Complete
.

CURRENT level of confidence

	I am not at all confident	I am a little confident	I am somewhat confident	I am quite confident	I am very confident
I am able to complete medical forms by myself	◯	◯	◯	◯	◯

Would it be easy for you to answer these questions via text?
◯ Yes
◯ No

Would it be easy for you to answer these questions via mobile app?
◯ Yes
◯ No

Best phone number
____

What is the best time of day to reach you?
◯ Morning 9am-12pm
◯ Afternoon 12pm-5pm
◯ Evening 5pm-8pm

Is there someone who know always knows how to get in touch with you?
◯ Yes
◯ No

If yes, please provide their phone number
____

What is your email address?
____

Phone number
____

What is your mailing address?
____

What is your preferred payment method?
◯ Venmo
◯ Cash App
◯ Virtual gift card

Gender
◯ Male
◯ Female
◯ Other

Race	◯ Non-Hispanic White<br>◯ Black or African-American<br>◯ Asian<br>◯ Other
Hispanic Ethnicity	◯ Hispanic<br>◯ Non-Hispanic
What is the highest degree or level of school you have completed?	◯ No schooling completed<br>◯ Grades 1 through 11<br>◯ High school diploma, GED or equivalent<br>◯ Some college, no degree<br>◯ Associates degree or certificate (for example: AA, AS)<br>◯ Bachelor's degree (for example: BA. BS)<br>◯ Master's degree (for example: MA, MS, MEng, MEd, MSW, MBA)<br>◯ Professional degree beyond bachelor's degree (for example: MD, DDS, DVM, LLB, JD)<br>◯ Doctorate degree (for example, PhD, EdD)
Are you currently employed?	◯ No<br>◯ Part-time (1 or more jobs)<br>◯ Full-time<br>◯ Retired

PROACT Follow-Up Survey

Please complete the survey below.

Thank you!

Patient Reported Outcomes in Acute Asthma Care and Treatment (PROAACT) Part B 7-Day Follow-Up Phone Survey

		Not at all	A little bit	Somewhat	Quite a bit	Very much
1)	In the last 7 days, how often was it easy to get the care, tests, or treatment you needed?	○	○	○	○	○
2)	In the last 7 days, how often were you unable to see a primary care doctor for your asthma because of the cost?	○	○	○	○	○
3)	In the last 7 days, how often were you referred to a specialist for asthma care but could not go because of the cost?	○	○	○	○	○
4)	In the last 7 days, how often was it easy to get the medication you needed?	○	○	○	○	○
5)	In the last 7 days, was there a time when you needed to buy medication for your asthma but could not because of the cost?	○	○	○	○	○

Please consider the NEXT 7 days and indicate:

		Not at all	A little bit	Somewhat	Quite a bit	Very much
6)	In the next 7 days, I anticipate difficulty scheduling a follow up appointment with my primary care or asthma provider	○	○	○	○	○
7)	In the next 7 days, I anticipate difficulty attending a follow up appointment with my primary care or asthma provider	○	○	○	○	○
8)	In the next 7 days, I anticipate difficulty obtaining my prescribed medications	○	○	○	○	○

Since your ED visit, over the PAST 7 days, how short of breath did you get with each of these activities?

		No shortness of breath	Mildly short of breath	Moderately short of breath	Severely short of breath	I did not do this in the past 7 days
9)	Dressing yourself without help	○	○	○	○	○
10)	Walking 50 steps/ paces on flat ground at normal speed without stopping	○	○	○	○	○
11)	Walking up 20 stairs (2 flights) without stopping	○	○	○	○	○
12)	Preparing meals	○	○	○	○	○
13)	Washing dishes	○	○	○	○	○
14)	Sweeping or mopping	○	○	○	○	○
15)	Making a bed	○	○	○	○	○
16)	Lifting something weighting 10-20 lbs (about 4.5-9kg, like a large bag of groceries)	○	○	○	○	○
17)	Carrying something weighing 10-20 lbs (about 4.5-9kg, like a large bag of groceries) from one room to another	○	○	○	○	○
18)	Walking (faster than your usual speed) for mile (almost 1 km or 10 blocks) without stopping	○	○	○	○	○

Please indicate you CURRENT level of confidence

		I am not at all confident	I am a little confident	I am somewhat confident	I am quite confident	I am very confident
19)	I can follow directions when my doctor changes my medications	○	○	○	○	○
20)	I can take my medication when there is a change in my usual day (unexpected things happen)	○	○	○	○	○
21)	I can manage my medications without help	○	○	○	○	○
22)	I can list my medications, including the doses and schedule	○	○	○	○	○

Please indicate your CURRENT level of confidence

		I am not at all confident	I am a little confident	I am somewhat confident	I am quite confident	I am very confident
23)	I can manage my symptoms during my daily activities	○	○	○	○	○
24)	I can keep my symptoms from interfering with relationships with friends and familty	○	○	○	○	○
25)	I can manage my symptoms in a public place	○	○	○	○	○
26)	I can work with my doctor to manage my symptoms	○	○	○	○	○

Since your ED visit, in the PAST 7 days:

		Never	Rarely	Sometimes	Usually	Always
27)	I have trouble doing all of my regular leisure activities with others	○	○	○	○	○
28)	I have trouble doing all of the family activities that I want to do	○	○	○	○	○
29)	I have trouble doing all of my usual work (include work at home)	○	○	○	○	○
30)	I have trouble doing all of the activities with friends that I want to do	○	○	○	○	○

Since your ED visit, in the PAST 7 days

		Not at all	A little bit	Somewhat	Quite a bit	Very much
31)	I am satisfied with how much work I can do (include work at home)	○	○	○	○	○
32)	I am satisfied with my ability to work (include work at home)	○	○	○	○	○
33)	I am satisfied with my ability to do regular personal and household responsibilities	○	○	○	○	○
34)	I am satisfied with my ability to perform my daily routines	○	○	○	○	○

Please indicate your CURRENT level of concern

		Not at all	A little bit	Somewhat	Quite a bit	Very much
35)	I am worried about returning to the ED in the next 7 days because my symptoms will continue or worsen	○	○	○	○	○
36)	I am worried about returning to the ED in the next 7 days because I won't be able to get my medications	○	○	○	○	○
37)	I am worried about returning to the ED in the next 7 days because I won't be able to see my doctor	○	○	○	○	○

Please indicate your CURRENT level of concern:

		Not at all	A little bit	Somewhat	Quite a bit	Very much
38)	I worry about becoming short of breath	○	○	○	○	○
39)	I become afraid when I have trouble catching my breath	○	○	○	○	○
40)	Exercise seems unsafe for me	○	○	○	○	○
41)	I am embarrassed by using my inhaled medication in public	○	○	○	○	○
42)	I am embarrassed by using my oxygen in public (skip if you don't use oxygen)	○	○	○	○	○
43)	I am embarrassed by my shortness of breath	○	○	○	○	○
44)	I get upset when I can't do something because of my shortness of breath	○	○	○	○	○
